# Red LED Treatment Confers Superior Early Post‐Thaw Sperm Kinetic Resilience and Identifies Progressive Motility as the Most Informative Functional Marker in Dogs

**DOI:** 10.1111/rda.70313

**Published:** 2026-08-23

**Authors:** Romulo Silva de Oliveira, Natália Santana Siqueira de Lara, Mayara Silvestri, Soraia Figueiredo de Souza, Luiz Ernandes Kozicki, Danilo Demeterco, Eduarda Stankiwich Vaz, Francielli Saczuk Rodrigues de Oliveira, Vivian Rotuno Moure, Glaucio Valdameri, Fernando Andrade Souza

**Affiliations:** ^1^ Universidade Federal Do Acre Rio Branco Acre Brazil; ^2^ Universidade Federal Do Paraná Curitiba Paraná Brazil; ^3^ Pontifícia Universidade Católica Do Paraná Curitiba Paraná Brazil; ^4^ Laboratory of Cancer Drug Resistance Federal University of Paraná Curitiba Paraná Brazil

**Keywords:** dog spermatozoa, photobiomodulation, progressive motility, semen cryopreservation, thermoresistance test

## Abstract

LED‐based photobiomodulation has attracted increasing interest as a potential strategy to improve post‐thaw sperm performance, but its biological relevance in canine semen remains poorly defined. This study investigated whether blue and red LED exposure influences baseline sperm quality and the dynamic pattern of post‐thaw sperm deterioration in dogs. Semen from six healthy adult dogs was collected, diluted, allocated to control (no LED), blue LED, or red LED groups, and cryopreserved under standardized conditions. After thawing, sperm quality was evaluated by computer‐assisted sperm analysis (CASA), hypo‐osmotic swelling test (HOST), eosin–nigrosin staining, and a rapid thermoresistance test. Relative motility retention at 10 min post‐thaw was calculated as a synthetic indicator of short‐term kinetic resilience. No significant baseline differences were detected among LED conditions for any seminal or CASA‐derived parameter. In contrast, post‐thaw sperm kinetics were clearly time‐dependent and treatment‐sensitive. Total motility showed a strong temporal decline and a significant treatment‐dependent decay pattern, whereas progressive motility exhibited significant effects of treatment, time, and treatment × time interaction, indicating greater sensitivity for detecting functional differences among LED conditions. The red LED condition preserved a substantially greater fraction of both total and progressive motility during the first 10 min after thawing, demonstrating superior short‐term post‐thaw kinetic resilience. In conclusion, red LED was the most effective condition for preserving early post‐thaw sperm kinetic resilience in canine semen, and progressive motility proved to be the most sensitive functional marker for detecting treatment‐related differences.

## 1 Introduction

Canine semen cryopreservation is an important reproductive biotechnology for genetic conservation, international germplasm exchange, and the clinical management of valuable sires. However, despite its practical relevance, freezing and thawing still impose substantial injury to sperm cells, compromising motility, membrane integrity, and overall functional competence after thawing. Cold shock, osmotic stress, ice crystal formation, and oxidative imbalance have all been implicated in the deterioration of sperm performance during cryopreservation, making post‐thaw quality one of the main limiting factors for the broader efficiency of this technology in dogs (Eilts 2005; Bencharif and Dordas‐Perpinya 2020; Sicherle et al. 2020; Axnér 2026).

Among post‐thaw endpoints, sperm motility remains one of the most informative and routinely applied indicators of functional quality. In canine semen, both total and progressive motility are directly affected by the freezing–thawing process, and CASA‐based analysis has become especially useful for detecting quantitative and qualitative changes in sperm motion patterns after thawing. Importantly, baseline semen quality does not always accurately predict post‐thaw performance, which reinforces the need to investigate not only static seminal characteristics but also dynamic functional behavior during the early post‐thaw period (Cardoso et al. 2007; Schäfer‐Somi and Tichy 2019; Christensen and Meyers 2023).

In recent years, photobiomodulation has attracted increasing interest as a strategy to modulate sperm physiology. Experimental studies and systematic reviews suggest that exposure to visible or near‐infrared light, particularly within the red spectrum, may enhance sperm energetic status, mitochondrial activity, and motility without inducing detectable cellular damage when appropriately dosed (Preece et al. 2017; Poorhassan et al. 2022; Moradi et al. 2024; Balbi et al. 2025). In sperm cells, this effect is biologically plausible because motility is highly dependent on mitochondrial energy metabolism, ATP availability, flagellar coordination, calcium signalling, nitric oxide modulation, and the balance between physiological and excessive reactive oxygen species production. Red and near‐infrared wavelengths have been proposed to interact with mitochondrial photoacceptors, especially cytochrome c oxidase, thereby influencing electron transport, mitochondrial membrane activity, ATP generation, and redox signalling. These mechanisms may be particularly relevant after thawing, when spermatozoa are exposed to abrupt metabolic, osmotic, oxidative, and membrane‐related stressors that compromise the maintenance of progressive movement over time (Preece et al. 2017; Saylan et al. 2023; Ahmed et al. 2024; Moradi et al. 2024; Balbi et al. 2025).

Nevertheless, the potential relevance of LED exposure for frozen–thawed canine semen remains insufficiently characterized. In particular, it remains unclear whether different LED conditions influence only baseline seminal characteristics or whether their biological effect becomes more evident during the early dynamic phase of post‐thaw sperm deterioration. This distinction is important because an intervention may fail to produce marked differences at baseline while still exerting a meaningful effect on short‐term sperm kinetic resilience after thawing. From a functional perspective, this issue is especially relevant when sperm performance is assessed longitudinally during thermoresistance testing rather than exclusively at a single post‐thaw time point.

Therefore, the present study was designed to determine whether LED condition modifies not only baseline sperm characteristics but also the dynamic pattern of post‐thaw sperm functional decline in dogs. Special emphasis was placed on early kinetic resilience after thawing, with the a priori hypothesis that red LED exposure would better preserve post‐thaw sperm motility than blue LED or no LED exposure, and that progressive motility would better discriminate treatment‐related differences than total motility.

## 2 Materials and Methods

### 2.1 Ethical Approval and Study Setting

All animal procedures were conducted in accordance with the guidelines of the Brazilian National Council for the Control of Animal Experimentation (CONCEA) and were approved by the institutional ethics committee (protocol no. 067/2024). The study was carried out in Curitiba, Paraná, Brazil.

### 2.2 Animals, Semen Collection, and Initial Processing

Six healthy adult mixed‐breed male dogs, weighing 12–15 kg and considered suitable for reproduction, were included in the study. Semen was collected by digital manipulation, and only the sperm‐rich fraction was used for subsequent processing. Immediately after collection, ejaculates were evaluated for initial quality and diluted to a final concentration of 100 × 10^6^ sperm/mL in a Tris‐based extender supplemented with egg yolk and 4% glycerol. At the time of dilution, the extender was maintained at 37°C. After dilution, each ejaculate was divided into three paired aliquots corresponding to the experimental conditions: control, blue LED, and red LED. This within‐ejaculate design was used to minimize inter‐individual variation and to allow direct comparison among treatments within the same ejaculate. The animals exhibited an initial motility of at least 70%. Animals with motility below the pre‐established threshold were not accepted; the mean and standard deviation were 75.33 ± 6.26.

The animals exhibited an initial motility of at least 70%. Animals with motility below the pre‐established threshold were not accepted; the mean and standard deviation were 75.33 ± 6.26.

### 2.3 Experimental Design and LED Treatments

Each diluted ejaculate was allocated to three paired experimental conditions: control, blue LED, and red LED. The LED irradiation system was custom‐built and generated peak wavelengths of 440 nm for blue light and 645 nm for red light. For this experimental phase, a light‐emitting diode‐based photodynamic therapy device was used, as previously described by Zanzarini et al. (2023). The spectral emission profile of the LED system was documented to confirm the peak wavelengths used for semen irradiation.

For LED exposure, semen aliquots were packaged into 0.5‐mL straws to ensure sample homogenization and standardized exposure conditions. Each straw contained 0.5 mL of diluted semen. The samples were positioned at a fixed distance of 225 mm from the LED source. Apart from the straw wall itself, no additional lid, glass plate, plastic cover, or optical barrier was placed between the LED source and the semen samples.

The irradiation protocol consisted of 6 min of continuous exposure, corresponding to a total irradiation dose of 1.638 J/cm^2^ at an energy delivery rate of 4.55 mJ/cm^2^/s. The blue LED group was exposed to 440 nm light, whereas the red LED group was exposed to 645 nm light. The control aliquot was maintained for the same 6‐min period under identical handling and temperature conditions but without LED exposure.

During irradiation, the LED device was placed inside an incubator maintained at 37°C to ensure controlled temperature conditions throughout exposure. All samples were protected from ambient light. The control group was kept in the dark under the same temperature and handling conditions, whereas the LED‐treated groups were exposed only to their assigned wavelength. No sperm motility assessment was performed immediately after LED exposure and before cryopreservation; the first post‐treatment motility evaluation was performed after thawing.

Following LED exposure, all aliquots from the same ejaculate were subjected to identical cooling and freezing procedures. The three experimental groups from each ejaculate were cooled and frozen simultaneously, characterizing a randomized block design in which the ejaculate served as the block.

### 2.4 Cryopreservation Procedure

After LED treatment, semen straws were subjected to a controlled cooling and freezing protocol. Samples were cooled using a BotuFlex cooling box, which allowed the temperature to decrease to 5°C over approximately 1 h. After reaching 5°C, the samples were maintained under these cooling conditions for 4 h before freezing. For freezing, straws were exposed to liquid nitrogen vapour in a 27‐L Styrofoam box. A 5‐cm column of liquid nitrogen was used, and the 0.5‐mL straws were positioned approximately 3 cm above the liquid nitrogen surface for 15 min. After this vapour‐freezing step, the straws were plunged directly into liquid nitrogen and stored at −196°C until thawing. All treatment groups derived from the same ejaculate were cooled, equilibrated, frozen, and stored under the same conditions.

For post‐thaw evaluation, straws were thawed in a water bath at 37°C for 30 s. After thawing, samples were immediately processed for CASA evaluation, conventional sperm assessments, and the rapid thermoresistance test according to the experimental design.

### 2.5 Post‐Thaw Sperm Evaluation

After thawing, sperm quality was assessed using computer‐assisted sperm analysis (CASA) and conventional laboratory tests. CASA evaluation included total motility, progressive motility, curvilinear velocity (VCL), average path velocity (VAP), straight‐line velocity (VSL), straightness (STR), linearity (LIN), amplitude of lateral head displacement (ALH), and beat‐cross frequency (BCF), using a commercial system (SpermVision SAR, Minitube, Germany).

Additional functional assessments included the hypo‐osmotic swelling test (HOST), used as an indicator of plasma membrane functionality, and eosin–nigrosin staining, used to assess sperm viability. These evaluations were performed immediately after thawing in a water bath at 37°C for 30 s. These tests were included to provide an integrated evaluation of sperm motility and membrane‐related functional status after thawing.

### 2.6 Rapid Thermoresistance Test

To investigate short‐term post‐thaw sperm resilience, a rapid thermoresistance test was performed after thawing. Samples from each treatment group were incubated at 37°C, and total and progressive motility were assessed at 0 and 10 min after thawing.

The thermoresistance test was used to characterize the early temporal pattern of sperm kinetic deterioration under each LED condition. Particular emphasis was placed on total and progressive motility changes between 0 and 10 min, as well as on relative motility retention during the early post‐thaw interval. Relative motility retention at 10 min was calculated as the percentage of motility preserved at 10 min relative to the corresponding baseline value at 0 min within each treatment group.

### 2.7 Statistical Analysis

Results are presented as mean ± standard deviation (SD). The ejaculate was considered the experimental unit. Because each ejaculate was divided into three paired aliquots corresponding to the control, blue LED, and red LED conditions, the experimental design was treated as paired within ejaculate. Baseline post‐thaw seminal quality and CASA‐derived kinetic parameters were compared among LED conditions using Friedman's repeated‐measures test. Absolute total and progressive motility values obtained during the rapid thermoresistance test were also compared among LED conditions within each incubation time using Friedman's repeated‐measures test.

Relative motility retention at 10 min was calculated for each ejaculate and LED condition according to the following formula:

$\begin{array}{c} \text{Relative motility retention}\;(\left(\%\right)=\text{motility}\;\mathrm{at}\;10\;\min/\text{motility}\;\mathrm{at} \end{array}$ $\begin{array}{c} 0\;\min)\times 100 \end{array}$

This calculation was performed separately for total motility and progressive motility. Relative retention values were compared among LED conditions using Friedman's repeated‐measures test. When a significant global effect was detected, pairwise post hoc comparisons among LED conditions were performed using Wilcoxon signed‐rank tests with Holm adjustment for multiple comparisons. Different superscript lowercase letters were used in the tables to indicate significant differences among LED conditions within the same endpoint. When no significant pairwise differences were detected, no superscript letters were added. Differences were considered statistically significant at *p* < 0.05. All statistical analyses were performed using R software, version 4.5.3 (R Foundation for Statistical Computing, Vienna, Austria). Friedman's repeated‐measures tests, Wilcoxon signed‐rank tests, and Holm‐adjusted post hoc comparisons were performed using the stats package, version 4.5.3.

## 3 Results

### 3.1 Baseline Post‐Thaw Sperm Quality Before Thermoresistance Testing

Baseline post‐thaw sperm quality and CASA‐derived kinetic parameters were compared among LED conditions before the rapid thermoresistance test to determine whether the experimental groups were comparable at the start of the post‐thaw incubation period. No significant differences were detected among the control, blue LED, and red LED conditions for total motility, progressive motility, sperm viability assessed by eosin–nigrosin staining, plasma membrane functionality assessed by HOST, or any CASA‐derived kinetic variable (Table 1). Therefore, the three LED conditions showed comparable baseline post‐thaw sperm quality before the longitudinal thermoresistance evaluation.

**TABLE 1:** Baseline post‐thaw seminal and CASA‐derived kinetic parameters before the rapid thermoresistance test in frozen–thawed canine semen according to LED condition.

Parameter	Control	Blue LED	Red LED	*p*
Total motility (%)	33.16 ± 4.74	29.12 ± 5.36	30.39 ± 5.95	0.311
Progressive motility (%)	18.45 ± 3.56	15.67 ± 2.98	14.81 ± 4.90	0.311
Eosin–nigrosin (%)	74.50 ± 7.29	62.00 ± 18.19	58.17 ± 23.98	0.311
HOST (%)	53.17 ± 6.88	50.50 ± 15.18	44.50 ± 18.89	0.154
VCL (μm/s)	107.43 ± 19.93	102.09 ± 14.67	88.85 ± 30.42	0.115
VAP (μm/s)	66.64 ± 11.08	62.97 ± 8.16	54.24 ± 16.62	0.069
VSL (μm/s)	51.55 ± 7.94	49.23 ± 6.02	41.89 ± 12.42	0.069
STR (%)	71.36 ± 16.64	71.83 ± 16.35	71.40 ± 16.92	0.135
LIN (%)	44.70 ± 11.19	44.81 ± 11.13	44.69 ± 12.17	0.311
ALH (μm)	3.08 ± 0.72	2.94 ± 0.55	2.55 ± 0.99	0.115
BCF (Hz)	13.91 ± 3.22	13.21 ± 2.40	11.52 ± 4.48	0.115

*Note:* Values are presented as mean ± SD. *p*‐values were obtained using Friedman's repeated‐measures test, consistent with the paired within‐ejaculate design.

Abbreviations: ALH, amplitude of lateral head displacement; BCF, beat‐cross frequency; CASA, computer‐assisted sperm analysis; HOST, hypo‐osmotic swelling test; LIN, linearity; STR, straightness; VAP, average path velocity; VCL, curvilinear velocity; VSL, straight‐line velocity.

### 3.2 Absolute Total and Progressive Motility During the Rapid Thermoresistance Test

To evaluate whether LED condition influenced early post‐thaw sperm kinetic behaviour, total and progressive motility were analysed during the rapid thermoresistance test. Absolute motility values were compared among LED conditions at each incubation time, allowing direct assessment of treatment‐related differences at the beginning and at the end of the post‐thaw incubation period.

Total and progressive motility declined during the thermoresistance test in all experimental conditions. At 0 min, no significant differences were detected among LED conditions, indicating comparable baseline post‐thaw motility before incubation. At 10 min, the red LED group showed the highest numerical values for both total and progressive motility, whereas the control group showed an almost complete loss of sperm movement. Thus, the main treatment‐related signal was associated with motility preservation during early post‐thaw incubation rather than with superior baseline motility.

### 3.3 Relative Post‐Thaw Motility Retention

To complement the analysis of absolute motility values, short‐term sperm kinetic resilience was assessed by calculating the percentage of motility retained at 10 min relative to the corresponding baseline value at 0 min within each LED condition. This analysis allowed treatment‐related differences in motility preservation to be evaluated independently of baseline post‐thaw motility.

Relative retention of both total and progressive motility differed among LED conditions. Pairwise post hoc comparisons indicated that the red LED group preserved a significantly greater fraction of baseline motility than both the control and blue LED conditions for both endpoints. The control group showed almost no preservation of baseline motility at 10 min. These findings indicate that the most relevant treatment‐related effect was the preservation of sperm movement during early post‐thaw incubation rather than an immediate increase in baseline motility.

**FIGURE 1:**
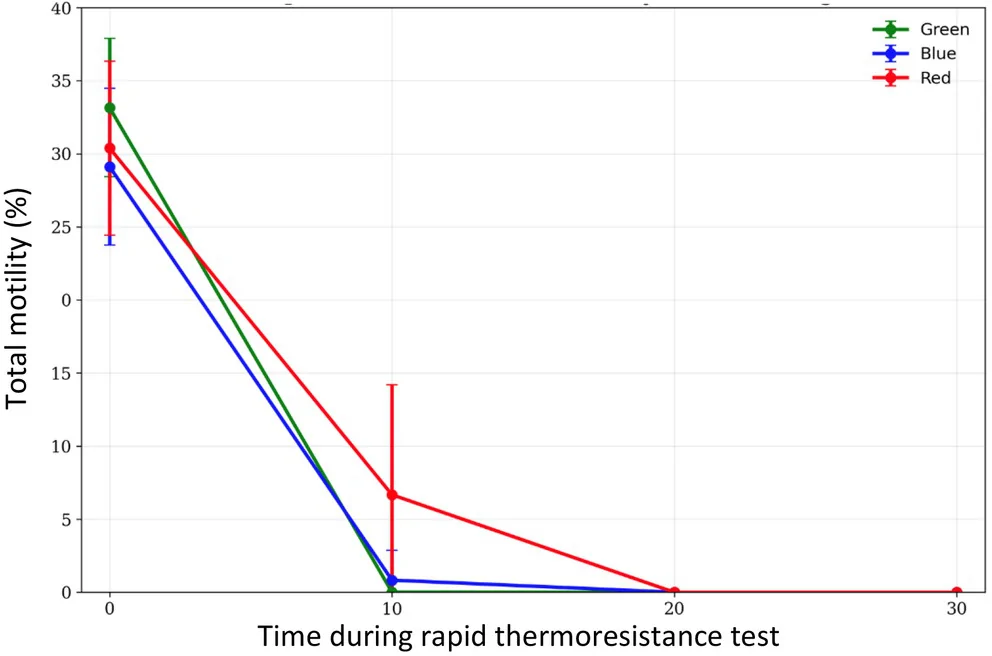
Total motility during the rapid thermoresistance test in frozen–thawed canine semen according to LED condition. Values are presented as mean ± SD. Control, blue LED, and red LED conditions are represented according to the colour legend shown in the figure.

**FIGURE 2:**
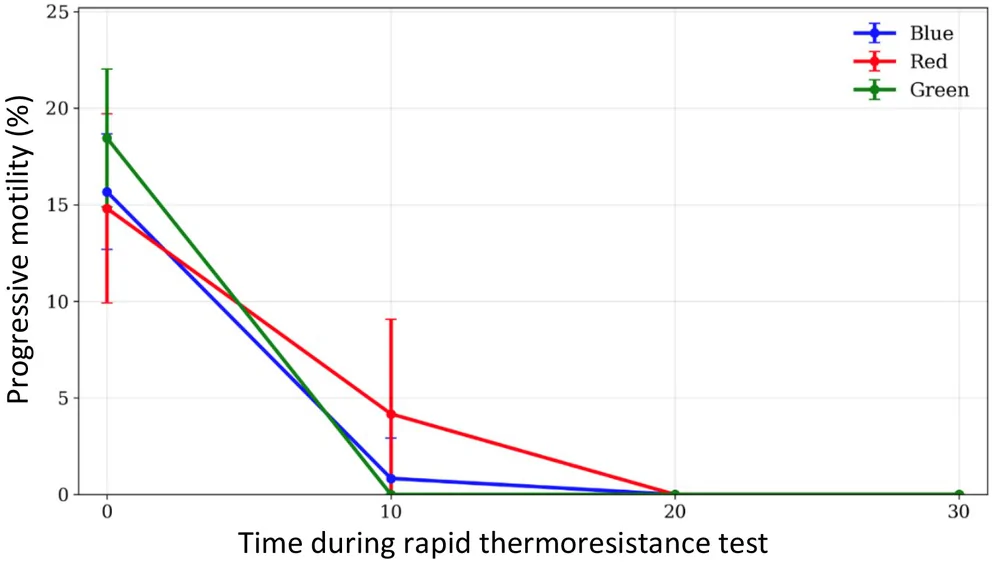
Progressive motility during the rapid thermoresistance test in frozen–thawed canine semen according to LED condition. Values are presented as mean ± SD. Control, blue LED, and red LED conditions are represented according to the colour legend shown in the figure.

**FIGURE 3:**
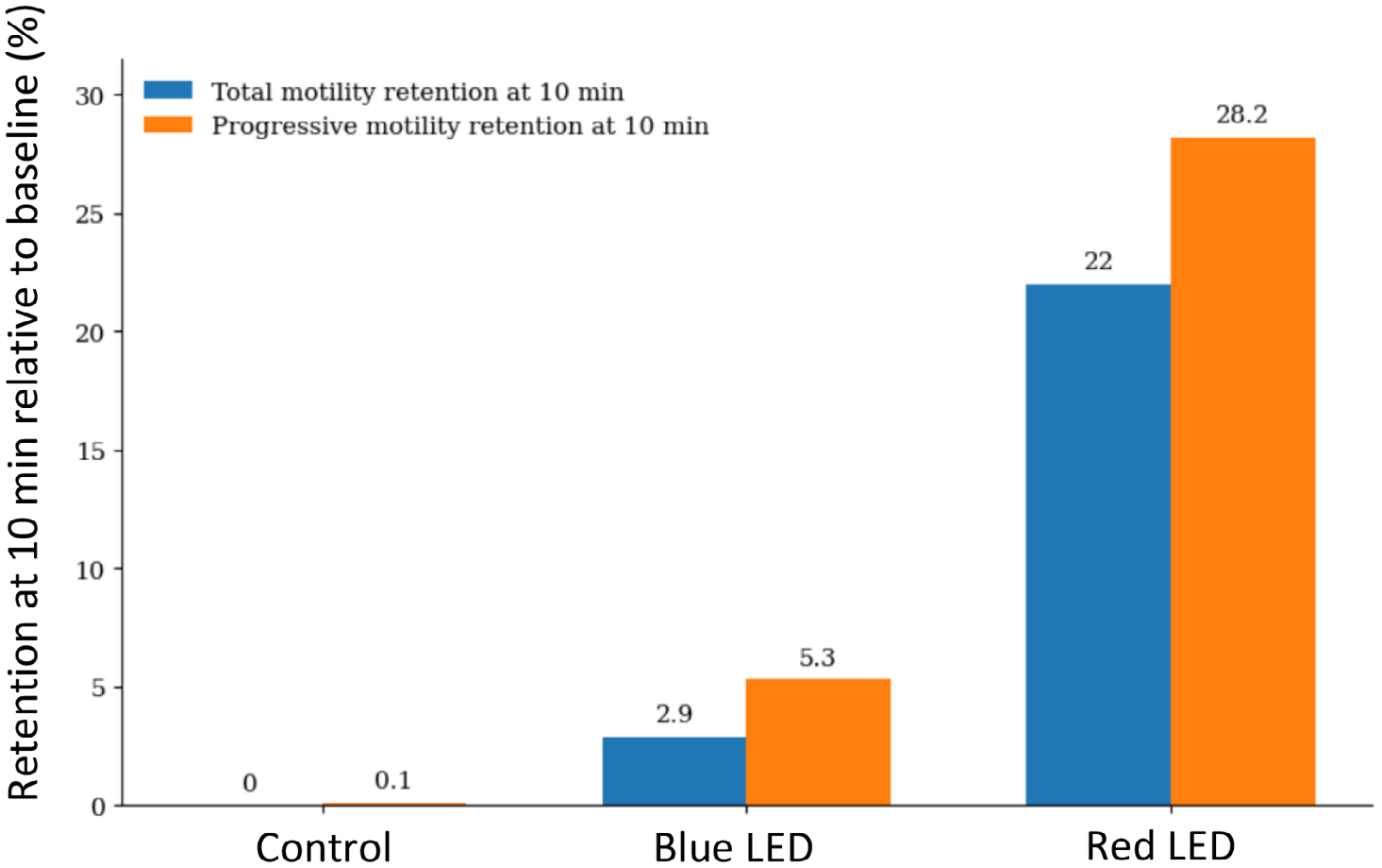
LED irradiation system used for the exposure of semen prior to cryopreservation. Blue and red LEDs were used, with wavelengths of 440 and 645 nm, respectively.

Post‐thaw sperm kinetic behaviour under the different LED conditions is illustrated in Figures 1 and 2. Figure 1 shows the temporal pattern of total motility during the rapid thermoresistance test, whereas Figure 2 shows the corresponding pattern for progressive motility. The spectral emission profile of the custom‐built LED irradiation system used before cryopreservation is shown in Figure 3.

Total motility decreased during the rapid thermoresistance test in all experimental conditions, with the red LED group showing higher numerical motility values during the early post‐thaw interval.

Progressive motility followed a similar temporal decline, but the red LED group maintained higher numerical values during the early post‐thaw interval, supporting the interpretation that progressive motility is a sensitive functional marker of post‐thaw sperm kinetic resilience.

The spectral profile confirms the wavelength separation between the blue and red LED conditions used for semen irradiation before cryopreservation.

## 4 Discussion

This study demonstrated that LED condition did not produce significant baseline differences in conventional seminal traits or in the main CASA‐derived variables before the rapid thermoresistance test (Table 1), but did influence relative early post‐thaw motility preservation, as demonstrated by the retention analysis (Table 3). Absolute motility values during the thermoresistance test are presented in Table 2 and illustrated in Figures 1 and 2. In canine semen, cryopreservation injury is not expressed only as an immediate reduction in quality at thawing, but also as a loss of post‐thaw functional longevity, particularly in motility‐related endpoints. Freezing and thawing expose spermatozoa to cold shock, membrane phase transitions, osmotic imbalance, and oxidative stress, all of which compromise sperm movement and membrane integrity (Lucio et al. 2016; Sicherle et al. 2020; Qamar et al. 2020; Axnér 2026).

**TABLE 2:** Total and progressive motility during the rapid thermoresistance test in frozen–thawed canine semen according to LED condition and incubation time.

Endpoint	Time	Control	Blue LED	Red LED	*p* among groups
Total motility (%)	0 min	33.16 ± 4.74	29.12 ± 5.36	30.39 ± 5.95	0.311
Total motility (%)	10 min	0.01 ± 0.02	0.83 ± 2.04	6.67 ± 7.52	0.058
Progressive motility (%)	0 min	18.45 ± 3.56	15.67 ± 2.98	14.81 ± 4.90	0.311
Progressive motility (%)	10 min	0.01 ± 0.02	0.83 ± 2.04	4.17 ± 4.91	0.058

*Note:* Values are presented as mean ± SD. *p*‐values compare LED conditions within each incubation time and were obtained using Friedman's repeated‐measures test, consistent with the paired within‐ejaculate design.

Abbreviation: LED, light‐emitting diode.

Within this context, the absence of significant baseline differences among control, blue LED, and red LED conditions (Table 1) should not be interpreted as evidence of biological irrelevance of the LED treatments. Rather, it indicates that the effect of LED exposure became more evident under post‐thaw functional challenge, when sperm cells were required to maintain coordinated movement over time (Tables 2 and 3; Figures 1 and 2). A treatment may fail to generate large static differences immediately after thawing while still altering the preservation of motility during the early post‐thaw interval, which is often the more meaningful biological outcome in cryobiology (Qamar et al. 2020; Axnér 2026).

**TABLE 3:** Relative post‐thaw motility retention at 10 min in frozen–thawed canine semen according to LED condition.

LED condition	Total motility retention at 10 min (% of baseline)	Progressive motility retention at 10 min (% of baseline)
Control	0.03 ± 0.06ᵇ	0.05 ± 0.11ᵇ
Blue LED	3.56 ± 8.72ᵇ	6.41 ± 15.69ᵇ
Red LED	21.55 ± 25.60ᵃ	24.77 ± 30.66ᵃ
Global *p*‐value	0.032	0.032

*Note:* Values are presented as mean ± SD. Retention values were calculated as: motility at 10 min/motility at 0 min × 100. Global *p*‐values were obtained using Friedman's repeated‐measures test, consistent with the paired within‐ejaculate design. Pairwise post hoc comparisons among LED conditions were performed using Wilcoxon signed‐rank tests with Holm adjustment. Different superscript lowercase letters within the same column indicate significant differences among LED conditions.

A major finding of this study was that the red LED condition was associated with superior short‐term preservation of sperm motility, particularly when sperm performance was expressed as relative retention at 10 min (Table 3). In the revised analysis, pairwise post hoc comparisons indicated that red LED preserved a significantly greater fraction of baseline total and progressive motility than both the control and blue LED conditions. This reflects not merely higher absolute motility values in one treatment group, but greater kinetic resilience during the earliest post‐thaw interval. Such an interpretation is compatible with the broader photobiomodulation literature. Across species and experimental models, exposure to light, especially within the red and near‐infrared range, has been associated with improved sperm motility and velocity, although responses are highly dependent on wavelength, dose, irradiance, and exposure duration (Moradi et al. 2024; Saylan et al. 2023; Ahmed et al. 2024; Balbi et al. 2025).

The apparent benefit of the red LED condition in terms of short‐term kinetic preservation (Tables 2 and 3; Figures 1 and 2) is also mechanistically plausible. Photobiomodulation has been linked to modulation of mitochondrial respiration, ATP production, nitric oxide signalling, intracellular calcium dynamics, and reactive oxygen species balance. The spectral emission profile of the LED system confirmed distinct wavelength peaks for the blue and red conditions (Figure 3), which is relevant because sperm responses to photobiomodulation are strongly wavelength‐dependent. Because flagellar beating is highly energy‐dependent and closely coupled to mitochondrial function, even subtle photobiomodulatory effects may become functionally visible as improved maintenance of sperm motion during post‐thaw incubation rather than as marked differences at baseline (Tables 2 and 3; Figures 1 and 2) (Ahmed et al. 2024; Moradi et al. 2024; Balbi et al. 2025).

Nonetheless, the present data should not be overinterpreted. The results support a functional advantage in early post‐thaw kinetic preservation, not a generalized claim that red LED improved all aspects of seminal quality. Baseline seminal traits did not differ significantly among treatments (Table 1), and not all evaluated variables showed clear separation among conditions. In addition, VAP and VSL were numerically lower in the red LED group at baseline, although these differences were not statistically significant. Therefore, the subsequent advantage of red LED in relative motility retention should be interpreted as improved preservation of sperm movement during the thermoresistance test, rather than as evidence of superior initial sperm velocity. The most accurate interpretation is therefore narrower: red LED attenuated the early post‐thaw kinetic decline as demonstrated by relative motility retention at 10 min (Table 3), rather than broadly transforming baseline sperm characteristics. This framing is also more consistent with the photobiomodulation literature, which repeatedly emphasizes that sperm responses to light are highly protocol‐dependent and cannot be generalized across irradiation conditions (Moradi et al. 2024; Ahmed et al. 2024).

Another important result was that progressive motility remained a highly informative functional endpoint for detecting treatment‐related differences. Total motility provides a broad indication of whether sperm are moving but does not discriminate between cells with effective forward displacement and those exhibiting non‐progressive or oscillatory motion. Progressive motility captures a more demanding aspect of sperm performance, namely the ability to sustain coordinated forward movement, which depends on flagellar coordination and adequate bioenergetic support. Because of this dependency, progressive motility is often more sensitive to subtle mitochondrial and membrane‐related dysfunction than total motility. In the present study, relative retention of both total and progressive motility was significantly higher in the red LED group than in the control and blue LED groups, but progressive motility provides a stricter biological interpretation because it reflects preservation of forward‐moving spermatozoa during the early post‐thaw interval (Table 3) (Saylan et al. 2023; Ahmed et al. 2024).

The patterns observed for the control and blue LED conditions also deserve careful interpretation. The absence of a meaningful advantage for either of these conditions across the retention analysis indicates that, under the tested irradiation protocol, blue LED did not improve early post‐thaw motility preservation when compared with the control condition (Table 3; Figures 1 and 2). This is consistent with the available literature, which shows marked heterogeneity across wavelengths, light sources, doses, and biological models, and a stronger evidence base for red and near‐infrared wavelengths than for blue or control conditions (Moradi et al. 2024).

The lack of significant baseline differences in HOST and eosin–nigrosin (Table 1), combined with the stronger signal detected in motility retention (Table 3; Figures 1 and 2), suggests that the tested LED protocol acted more clearly on functional persistence than on gross structural markers detectable immediately after thawing. This does not imply that membrane biology was irrelevant. Canine cryopreservation injury is strongly linked to membrane destabilization, lipid reorganization, and ROS‐mediated damage, all of which can subsequently manifest as reduced motility and shorter functional lifespan (Lucio et al. 2016; Sicherle et al. 2020; Qamar et al. 2020; Zduńczyk et al. 2025). Rather, the present data indicate that these downstream consequences were captured more effectively through early post‐thaw kinetic behaviour and relative motility retention than through purely static baseline assessments.

From an applied standpoint, this distinction has practical relevance for canine reproduction. Frozen–thawed dog semen has inherently lower functional longevity than fresh semen, and its successful use often depends on precise reproductive timing. A treatment capable of preserving motility even within a relatively short post‐thaw window may therefore carry biological significance. The present results suggest that assessing LED effects exclusively at baseline (Table 1) would underestimate their potential relevance, since the main treatment‐related signal emerged during the early post‐thaw kinetic response (Tables 2 and 3; Figures 1 and 2). A framework centered on relative motility retention during thermoresistance testing, particularly for progressive motility, may be more appropriate for detecting functionally relevant treatment effects in canine semen cryobiology (Axnér 2026).

The present findings should be interpreted within the biological and analytical scope of the study. The experimental design was inherently more sensitive to treatment‐related differences in early post‐thaw kinetic behaviour than to subtle variation in secondary static endpoints. Accordingly, the absence of significant separation in some baseline variables should be interpreted cautiously rather than as definitive evidence of equivalence among LED conditions. In addition, the study was designed to assess post‐thaw sperm function in vitro, which precludes direct conclusions regarding fertilizing capacity or pregnancy outcome. Although the observed response pattern is compatible with photobiomodulation‐related modulation of bioenergetic and redox pathways, the underlying mechanism was not directly tested, since mitochondrial membrane potential, intracellular ROS, lipid peroxidation, and sperm DNA integrity were not assessed in the present protocol. These variables should be prioritized in future studies aimed at refining LED‐based protocols for canine sperm cryopreservation (Moradi et al. 2024; Ahmed et al. 2024; Balbi et al. 2025).

The present findings should not be interpreted as a simplistic statement that one LED condition broadly improved semen quality. A more accurate and scientifically defensible interpretation is that LED condition influenced early post‐thaw sperm kinetic resilience, with red LED preserving a significantly greater fraction of baseline total and progressive motility than both the control and blue LED conditions (Table 3). This conclusion is grounded in sperm physiology, aligned with the known consequences of cryodamage, and consistent with the current photobiomodulation literature (Axnér 2026; Moradi et al. 2024; Ahmed et al. 2024).

## 5 Conclusions

The present study demonstrates that LED condition did not significantly affect baseline post‐thaw seminal quality but did influence short‐term sperm kinetic resilience during the rapid thermoresistance test. Red LED preserved a significantly greater fraction of baseline total and progressive motility at 10 min than both the control and blue LED conditions, indicating superior early post‐thaw motility preservation under the tested protocol. These findings suggest that the biological relevance of red LED exposure in canine semen cryopreservation lies not in altering baseline seminal characteristics, but in enhancing the maintenance of sperm movement during the early post‐thaw interval. Progressive motility retention appears to be a particularly informative functional marker for detecting treatment‐related differences in post‐thaw sperm resilience.

## Author Contributions

**Romulo Silva de Oliveira:** conceptualization, data curation, investigation, resources, writing – review and editing; **Natália Santana Siqueira de Lara:** formal analysis, writing – original draft, writing – review and editing; **Mayara Silvestri:** writing – original draft, writing – review and editing; **Soraia Figueiredo de Souza:** methodology, formal analysis, writing – review and editing; **Luiz Ernandes Kozicki:** methodology, formal analysis, writing – review and editing; **Danilo Demeterco:** methodology, formal analysis, writing – review and editing; **Eduarda Stankiwich Vaz:** validation, writing – review and editing. **Francielli Saczuk Rodrigues de Oliveira:** validation, writing – review and editing; **Vivian Rotuno Moure:** data curation, validation, writing – review and editing; **Glaucio Valdameri:** supervision, writing – review and editing; **Fernando Andrade Souza:** supervision, writing – review and editing.

## Conflicts of Interest

The authors declare no conflicts of interest.

## Data Availability

The data that support the findings of this study are available from the corresponding author upon reasonable request.
